# Nanozyme-driven multifunctional dressings: moving beyond enzyme-like catalysis in chronic wound treatment

**DOI:** 10.1186/s40779-025-00611-5

**Published:** 2025-05-31

**Authors:** Si-Jie Zhang, Ran Xu, Shao-Bin He, Rong Sun, Guan-Nan Wang, Shu-Yi Wei, Xi-Yun Yan, Ke-Long Fan

**Affiliations:** 1 CAS Engineering Laboratory for Nanozyme, Key Laboratory of Biomacromolecules (CAS), CAS Center for Excellence in Biomacromolecules, Institute of Biophysics, Chinese Academy of Sciences, Beijing, 100101 China; 2 College of Life Sciences, University of Chinese Academy of Sciences, Beijing, 101408 China; 3 Laboratory of Clinical Pharmacy, Department of Pharmacy, the Second Affiliated Hospital of Fujian Medical University, Quanzhou, 362000 Fujian China; 4 Department of Radiation Oncology, Jinling Hospital, Affiliated Hospital of Medical School, Nanjing University, Nanjing, 210000 China; 5 Shenyang Key Laboratory of Medical Molecular Theranostic Probes in School of Pharmacy, Shenyang Medical College, Shenyang, 110034 China; 6 Peking University People’s Hospital, Beijing, 100044 China; 7 Nanozyme Laboratory in Zhongyuan, Henan Academy of Innovations in Medical Science, Zhengzhou, 451163 China

**Keywords:** Nanozyme, Enzyme-like activities, Reactive oxygen species regulation, Chronic wound therapy, Multifunctional wound dressing

## Abstract

The treatment of chronic wounds presents significant challenges due to the necessity of accelerating healing within complex microenvironments characterized by persistent inflammation and biochemical imbalances. Factors such as bacterial infections, hyperglycemia, and oxidative stress disrupt cellular functions and impair angiogenesis, substantially delaying wound repair. Nanozymes, which are engineered nanoscale materials with enzyme-like activities, offer distinct advantages over conventional enzymes and traditional nanomaterials, making them promising candidates for chronic wound treatment. To enhance their clinical potential, nanozyme-based catalytic systems are currently being optimized through formulation advancements and preclinical studies assessing their biocompatibility, anti-oxidant activity, antibacterial efficacy, and tissue repair capabilities, ensuring their safety and clinical applicability. When integrated into multifunctional wound dressings, nanozymes modulate reactive oxygen species levels, promote tissue regeneration, and simultaneously combat infections and oxidative damage, extending beyond conventional enzyme-like catalysis in chronic wound treatment. The customizable architectures of nanozymes enable precise therapeutic applications, enhancing their effectiveness in managing complex wound conditions. This review provides a comprehensive analysis of the incorporation of nanozymes into wound dressings, detailing fabrication methods and emphasizing their transformative potential in chronic wound management. By identifying and addressing key limitations, we introduce strategic advancements to drive the development of nanozyme-driven dressings, paving the way for next-generation chronic wound treatments.

## Background

**Fig. 1:**
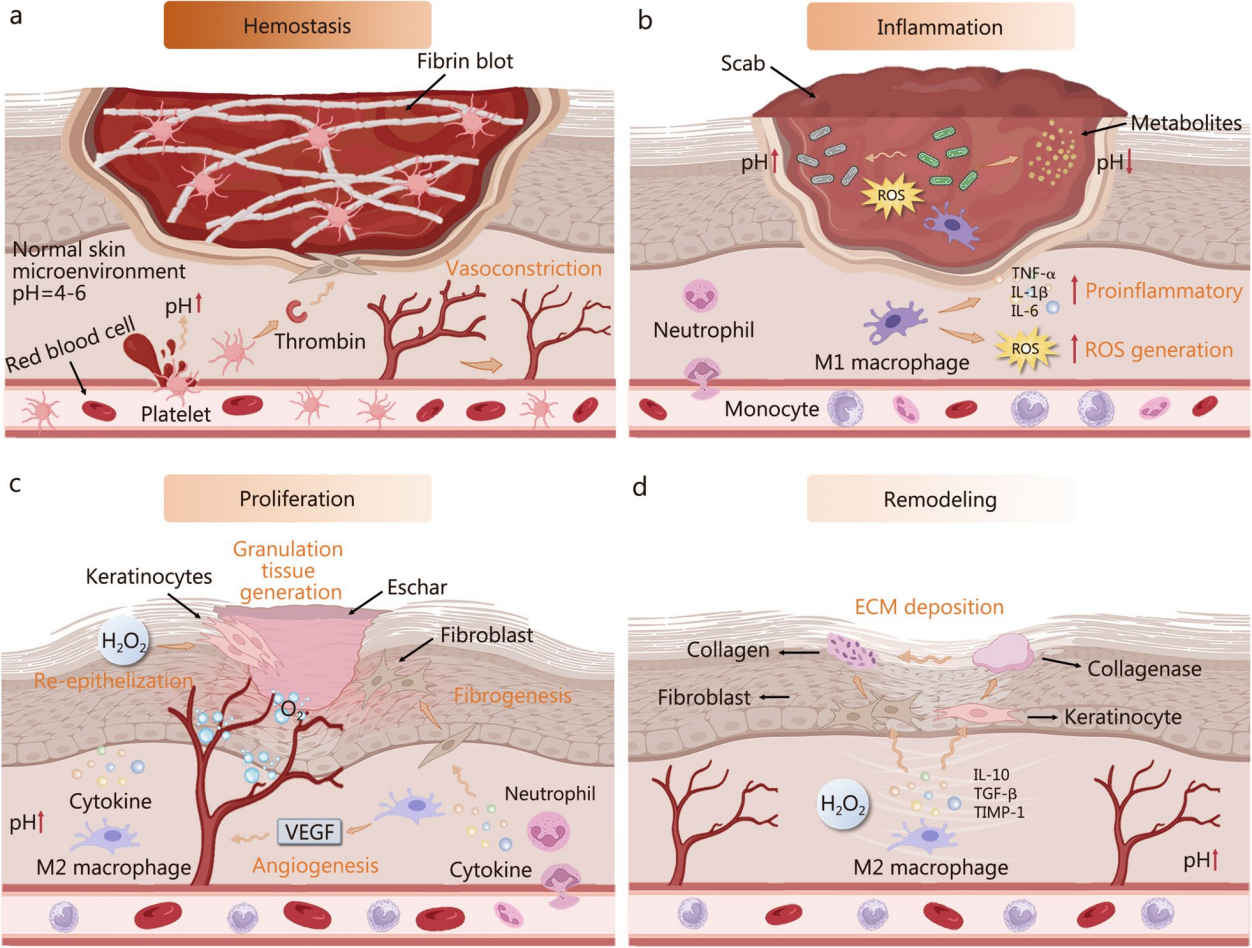
Overall wound healing progress. **a** Hemostasis begins with platelet aggregation to prevent blood loss, resulting in the formation of an initial fibrin framework.** b** The inflammatory phase is triggered by immune receptors that detect pathogens, leading to immune cell recruitment and pro-inflammatory signaling. **c** In the proliferative phase, fibroblasts, keratinocytes, and vascular endothelial cells form granulation tissue to promote extracellular matrix (ECM) remodeling. Meanwhile, macrophages secrete vascular endothelial growth factor (VEGF) to promote angiogenesis, facilitating the transport of oxygen to the wound site and recovering energy supply. **d** The remodeling phase is characterized by the continued synthesis and degradation of collagen in the ECM. Cytokines (especially TGF-β) stimulate fibroblasts to produce collagen, which is then broken down by enzymes to restore the normal structure of the dermis. TNF-α tumor necrosis factor-α, IL interleukin, ROS reactive oxygen species, TGF-β transforming growth factor-β, TIMP-1 tissue inhibitor of metallopeptidase-1

In medical terminology, wounds refer to sites of injury and tissue disruption, particularly in the skin, muscles, and mucosa. The standard process of wound healing encompasses four interconnected and successive phases: hemostasis, inflammation, proliferation, and remodeling [1, 2] (Fig. 1). However, disruptions in fundamental pathophysiological mechanisms or microbial intrusion can impair the normal reparative process, leading to chronic wounds. In immunocompromised individuals, particularly those with diabetes, neuropathy, or circulatory disorders, wound healing becomes prolonged and intricate. These conditions contribute to hypoxia, persistent inflammation, oxidative stress, and increased infection risk, collectively disrupting the wound microenvironment and forming a complex pathological network that hinders healing [3–9]. Restoring redox balance and inflammatory homeostasis in chronic wounds is essential for effective healing. While traditional debridement aids healing by removing necrotic tissue, it may be unsuitable for certain wounds, such as those caused by aortic insufficiency [10], and carries the risk of deeper infection [11]. While classic antibiotics and other local treatments remain widely used, they face growing challenges due to bacterial resistance. Bioengineered dermal substitutes offer potential benefits for wound healing, but they present a risk of immune rejection and fail to optimize the wound microenvironment [12]. Moreover, current therapeutic strategies for chronic wounds often target specific healing phases while overlooking the need for comprehensive wound environment modulation.

Nanozymes represent a novel class of nanomaterials that function as artificial enzymes, catalyzing substrate conversion while adhering to enzyme kinetics within a physiological environment. By integrating the unique properties of nanomaterials with catalytic capabilities, nanozymes offer a promising alternative to natural enzymes [13]. Unlike their biological counterparts, nanozymes exhibit enhanced catalytic stability under adverse conditions, thereby improving the consistency and efficacy of treatments [14]. The synthesis of nanozymes is relatively straightforward and well-established, with chemical synthesis, solution synthesis, and thermal decomposition being among the most commonly used methods. Additionally, nanozymes show significant advantages in terms of controllability, versatility, and biosafety. Compared with conventional nanomaterials, nanozymes possess enzyme-like characteristics, representing a new paradigm in catalytic therapy (Table 1).

**Table 1:** Advantages of nanozymes over enzymes and nanomaterials for the treatment of wound healing

Effectiveness	Nanozymes	Enzymes	Nanomaterials
Production	Various preparation methods with fewer byproducts	Complicated extraction and purification steps	Various preparation methods with different categories
Drug resistance	Less susceptible to drug resistance	Hard to develop drug resistance	Develop drug resistance if only used as nanocarriers
Controllability	Easy to control the performance by modifying the active group or changing the environment	Hard to modify based on the complex structure	Easy to control the performance by modifying the active group or changing the environment
Multifunction	Integration of diagnosis and catalytic treatment is possible in wound healing	Mainly relying on catalytic therapy and activity is sensitive to the wound environment	Integration of diagnosis and treatment with a relatively simple mechanism
Safety	Convenient to predict and evaluate the metabolism, distribution, toxicity, and adverse reactions in vivo	Suffer from uncertain efficacy and allergic reactions	Behave potential toxicity

Nanozymes offer significant advantages in controllability, versatility, and biosafety, making them a powerful tool in catalytic therapy. As biocatalysts, nanozymes modulate the chronic wound microenvironment, exerting enzyme-like effects that regulate biological processes involved in wound healing [15]. Moreover, engineered nanozymes can be tailored to adjust enzyme-like activities [16], allowing them to dynamically respond to changes in the wound-healing process and enhance therapeutic efficacy.

Nanozyme-based catalytic systems have demonstrated significant potential in chronic wound management by regulating the wound microenvironment. Researchers are actively optimizing nanozyme formulations and carrier designs to facilitate their transition to clinical applications [17]. Comprehensive preclinical studies assess their biocompatibility, anti-oxidant capacity, antibacterial properties, and tissue repair potential through in vitro experiments. Additionally, chronic wound animal models have been utilized to evaluate the efficacy, safety, pharmacokinetics, and long-term therapeutic effects, ensuring the reliability and viability of nanozymes in chronic wound treatment.

Beyond their catalytic properties, nanozymes exhibit multifunctional composite characteristics, making them compatible with traditional wound care strategies. In recent years, bioactive wound dressings have gained increasing attention in clinical practice due to their high biocompatibility, permeability, and wettability [18]. Nanozymes can be incorporated into dressing matrices through various physical and chemical methods, including physical entrapment, chemical crosslinking, self-assembly, and electrospinning. This integration significantly enhances drug delivery efficiency, antibacterial activity, and overall wound healing. Strategically engineered nanozyme-driven wound dressings are designed to align with the chronic wound microenvironment, offering a multifunctional therapeutic platform with substantial clinical potential.

Previous reviews have focused primarily on the theoretical mechanisms and enzyme-like activities of nanozymes in chronic wound treatment, with limited attention given to their practical applications in dressing designs and multifunctional platforms [19, 20]. This review aims to extend beyond enzyme catalysis by analyzing the complex wound microenvironment and the underlying mechanisms of chronic wound pathogenesis. We explore key barriers to healing and discuss how nanozyme-driven multifunctional dressings can be strategically designed to overcome these challenges. Moreover, we discuss the synthesis, mechanisms, and design principles of nanozyme-driven dressings, emphasizing their roles in regulating reactive oxygen species (ROS), modulating inflammation, and promoting tissue regeneration. We also assess current nanozyme-driven wound dressings and their preclinical applications, highlighting progress toward clinical translation. By addressing existing challenges and offering potential solutions, this review aims to inspire further research into nanozyme-driven therapies for chronic wound management.

## Chronic wound microenvironments

The skin acts as the body’s primary defense against external pathogens, playing a critical role in maintaining homeostasis and preventing microbial invasion. When the skin is damaged, blood vessels rupture, depriving subcutaneous tissues of essential nutrients and exposing them to reduced oxygen (O_2_) levels and oxidative stress. This compromised barrier enables pathogens to infiltrate open wounds, thereby initiating inflammation. Subsequent vascular hypoxia and cellular dysfunction further impair the tissue repair process, posing significant challenges in the treatment of chronic wounds. This section examines the microenvironmental and molecular factors that contribute to impaired wound healing and offers insights into mechanisms that may facilitate accelerated recovery.

### Persistent inflammation

Following ischemia, the second phase of the wound healing process is initiated, occurring within a dynamic and complex inflammatory tissue microenvironment. Under normal circumstances, inflammation gradually subsides, transitioning into the proliferation and remodeling stages. However, in chronic wounds, a persistent inflammatory state is maintained due to abnormal wound microenvironments, characterized by elevated inflammatory cytokines and reduced levels of healing mediators [21, 22].

During early inflammation, the innate immune system is activated, leading to the recruitment and accumulation of neutrophils, macrophages, lymphocytes, monocytes, and mast cells at the wound site, guided by gradients of inflammatory factors and chemokines [23]. These immune cells respond to pathogen- and danger-associated molecular patterns [24]. Upon arrival at the injury site, inflammatory cells secrete various signaling molecules, such as monocyte chemoattractant protein-1, leukotriene B4, gasdermin, elastase, and myeloperoxidase, which collectively regulate the immune response.

Innate immune cells deploy several antimicrobial strategies, including ROS generation, degranulation, and neutrophil extracellular trap formation [25, 26]. Neutrophils play a crucial role in regulating early inflammation; however, their timely clearance via apoptosis is critical for resolving inflammation. In cases where this process is disrupted, such as bacterial infection, neutrophil accumulation persists, leading to elevated levels of elastase and matrix metalloproteinases (MMPs) that degrade the extracellular matrix (ECM) and impair tissue repair [27].

Macrophages are essential in both inflammation regulation and tissue regeneration. They engulf pathogens and secrete cytokines and growth factors that promote cellular proliferation and wound restoration [28]. Macrophages are classified into two major phenotypes: M1 (pro-inflammatory) and M2 (anti-inflammatory/reparative). During normal wound healing, macrophages shift from M1 to M2 phenotype to regulate inflammation and promote tissue repair [29]. M1 macrophages secrete pro-inflammatory cytokines, including interleukin (IL)-1, tumor necrosis factor (TNF)-α, IL-6, and IL-12 [30]. Molecules such as glycoprotein non-metastatic melanoma B and MMPs are involved in modulating this phenotypic switch [31, 32].

In chronic wounds, persistent inflammation is often due to a failure in the M1-to-M2 macrophage transition, driven by excessive pro-inflammatory cytokine production and inefficient clearance of apoptotic neutrophils [33]. In diabetic wounds, elevated miRNA-21 expression in macrophages contributes to the sustained release of inflammatory mediators, including IL-1α, TNF-α, inducible nitric oxide synthase, IL-6, and IL-8, further promoting the M1 phenotype [34–36]. The significant shift from an anti-inflammatory to a pro-inflammatory phenotype disrupts the equilibrium between M1 and M2 macrophages, leading to a sustained inflammatory milieu.

The immune system thus plays a central role in coordinating chronic wound healing. In this context, stimulus-responsive wound dressings have shown promise due to their ability to release therapeutic agents in response to environmental changes [37, 38]. Nanozymes, in particular, exhibit stimuli-responsive catalytic activity under external conditions [39, 40], offering a new avenue for immune modulation.

ROS produced by immune cells is a key mediator in macrophages’ phenotypic switching [41–43]. At physiological levels, ROS facilitates autocrine/paracrine signaling involved in wound healing, including the release of growth factors [44] and the recruitment of immune cells to the wound site [45]. However, the excessive production of ROS initiates pathological signaling pathways, promoting the infiltration of polymorphonuclear neutrophils, which in turn stimulates the expression of inflammatory cytokines and the production of ROS [46]. High ROS production disrupts inflammatory microenvironmental homeostasis and is detrimental to the next stage of wound healing, whereas nanozymes can modulate ROS levels to restore immune balance and thereby promote wound healing progression [47].

In diabetic patients, hyperglycemia further complicates wound healing by impairing chemokine expression, which in turn hampers the timely clearance of neutrophils. Moreover, hyperglycemia results in the overproduction of ROS, heightening oxidative stress within the wound environment [48]. Additionally, hyperglycemia increases the expression of pro-inflammatory cytokines, including TNF-α, IL-1, and IL-6, thereby facilitating the polarization of the M1 macrophage phenotype and exacerbating inflammation. The resulting feedback loop of M1 macrophage infiltration and oxidative stress is a key factor in the chronicity of diabetic wounds [49, 50].

### Bacterial infection

As mentioned above, the integumentary system serves as the body’s primary barrier against external threats. It effectively mitigates physical injuries from environmental factors, including abrasions and trauma, while safeguarding against harmful chemicals and the intrusion of pathogens. However, when the skin is compromised, its protective function is diminished, increasing the susceptibility of the wound site to microbial infiltration. Microorganisms from the air, water, contaminated substances, or even the body’s flora can colonize the exposed wound surface [51].

Several factors create a favorable environment for microbial survival and proliferation in chronic wounds. These include an alkaline pH, hypoxia, the presence of toxins, and biofilm formation. While normal skin maintains a mildly acidic pH (4.0 − 6.0), chronic wounds often become alkaline (pH 7.3 − 8.9) due to tissue necrosis [52], thereby facilitating microbial colonization. Studies have shown that bacterial activity maintains this elevated pH, further enhancing conditions for biofilm formation [53–55]. Moreover, as microorganisms consume O_2_ during their growth, chronic wounds become increasingly hypoxic, limiting cellular energy production and impairing the activity of essential growth factors required for tissue regeneration [56].

Microbial components, such as cell wall fragments and metabolic byproducts, can act as antigens, triggering immune responses. Certain bacterial pathogens excrete toxins that specifically impair innate immune defenses by inhibiting the activity of macrophages or neutrophils. Furthermore, the lysis of gram-negative bacteria, either through immune attack or antimicrobial treatment, triggers the release of endotoxins. These endotoxins stimulate the production of pro-inflammatory mediators, such as TNF-α, which in turn upregulate MMPs, contributing to ECM degradation and delayed wound healing [57, 58].

Persistent microbial presence can escalate inflammation and tissue damage. If the immune system fails to eliminate the infection, monomicrobial or polymicrobial biofilms may develop at the wound site [59]. These biofilms are composed of microbial communities embedded within a self-produced extracellular polymeric matrix rich in polysaccharides, lipids, proteins, and nucleic acids [60]. This matrix forms a protective barrier that shields the microbes from immune surveillance and prolongs the inflammatory phase [61]. Compared to acute wounds, chronic wounds typically harbor a higher density of polymicrobial biofilms [7].

Biofilm-associated communities facilitate interspecies interactions, enabling horizontal gene transfer and promoting synergistic or competitive behaviors that contribute to antimicrobial resistance. If untreated, these biofilms can continuously release planktonic cells that may colonize nearby tissues or spread systemically via the vascular or lymphatic systems, ultimately leading to systemic infection [62, 63].

Patient-related factors also influence the risk and severity of chronic wound infections. Individuals with autoimmune diseases, such as lupus, often exhibit compromised skin integrity, increasing the likelihood of bacterial colonization [64]. In addition, aging alters wound healing mechanisms, including ECM synthesis, inflammatory response, and microvascular dynamics, further predisposing elderly individuals to chronic infection and delayed recovery [65].

### Hypoxia and angiogenesis disorders

O_2_ plays a pivotal role in both acute and chronic wound healing, participating in every stage of the healing process, including the immune response to bacterial invasion, cellular proliferation, re-epithelialization, angiogenesis, and collagen synthesis [66]. In aerobic respiration, O_2_ enables glucose oxidation to generate adenosine triphosphate, supplying the energy necessary for effective wound healing. However, tissue injury results in blood vessel rupture, leading to impaired O_2_ delivery and localized tissue hypoxia. Consequently, collagen synthesis is compromised, and the immune system’s ability to fight infection is weakened due to insufficient energy availability [67].

Chronic wound complications, such as diabetes mellitus, hypertension, and other systemic diseases, often result in atherosclerosis, characterized by thickened and hardened blood vessel walls, narrowed lumens, and restricted blood flow, thereby intensifying tissue hypoxia [68]. In addition, inflammation and microbial infections exacerbate hypoxia, creating a vicious cycle that further hinders wound healing [69]. Persistent hyperglycemia damages the microvasculature surrounding wounds, causing endothelial dysfunction, increased vascular permeability, blood leakage, and microthrombosis [70], which further reduces blood perfusion and worsens local hypoxia [71].

Under normal conditions, wound hypoxia activates hypoxia-inducible factor-1α (HIF-1α), which regulates angiogenesis-related genes and stimulates the production of angiogenic growth factors [72–74]. However, in chronic wounds, there is a significant accumulation of immune cells, particularly neutrophils and macrophages. These inflammatory cells utilize substantial amounts of O_2_ while executing their immune responses. Furthermore, the inflammatory mediators secreted by these cells, including TNF-α and IL-1, induce vasoconstriction and increase permeability, further impairing O_2_ transport [75]. Moreover, the microbial biofilm occupying the wound bed hosts a diverse range of microorganisms that deplete O_2_ through their metabolic processes. Notably, anaerobic bacteria thrive in these hypoxic conditions, exacerbating O_2_ deficiency at the wound site [76]. Hypoxia inhibits endothelial cell function, limits their responsiveness to growth factors, and disrupts their metabolism and energy supply [77]. In normal wound healing, M2 macrophages promote angiogenesis by secreting pro-angiogenic factors like vascular endothelial growth factor (VEGF) and transforming growth factor-β [78]. However, in chronic wounds, sustained M1 polarization reduces VEGF and fibroblast growth factor levels, while inflammatory mediators (e.g., TNF-α and IL-1) further suppress endothelial cell activity [79], collectively impairing neovascularization [80, 81]. Reduced macrophage levels also compromise the formation of vascularized granulation tissue, exacerbating vascular dysfunction [80].

Hypoxia, inflammation, and dysregulated metabolism in chronic wounds lead to excessive ROS accumulation, which is detrimental to angiogenesis. Elevated levels of ROS induce oxidative stress, ultimately leading to cellular dysfunction and apoptosis. Apoptotic cells release pro-inflammatory mediators, worsening inflammation and tissue damage [82]. In addition, excess ROS suppresses the activation of VEGF receptors and diminishes downstream signaling pathways, consequently hindering the response of endothelial cells and angiogenesis [72].

### Abnormal cell function during tissue regeneration

The initial proliferation phase involves complex changes in the inflammatory microenvironment and serves as a bridge for various cellular interactions, including immune cells, fibroblasts, and keratinocytes [83]. Alterations in mechanical forces and electrical potentials, along with exposure to hydrogen peroxide (H_2_O_2_), stimulate keratinocytes to move horizontally across the wound bed to reestablish the epidermis, a process known as re-epithelialization [84]. Keratinocytes secrete MMPs to facilitate their movement and contribute to the reconstruction of the basement membrane [85]. Fibroblasts play a central role in granulation tissue formation by replacing the provisional fibrin-rich matrix, acting as critical regulators of both physiological and pathological ECM deposition and remodeling. This transition provides a more substantial structural platform for the wound to proceed toward the final stages of repair [86]. While the proliferation stage emphasizes cellular activity, the remodeling phase focuses on molecular restructuring, particularly of the ECM. It extends from the initial stage of fibrin clot formation to the final stage of mature scar formation and is characterized by a rich presence of type I collagen [87], which enhances tissue strength and structural integrity.

As previously stated, in chronic wounds, sustained inflammation and redox imbalance impair both the proliferation and remodeling phases. A key pathological feature is the failure of macrophage phenotypic transition, which subsequently hampers the migration, proliferation, and differentiation of keratinocytes and fibroblasts within the wound bed [88]. Inflammation-induced ROS activate NF-κB, upregulating MMP-9. While physiological MMP-9 aids ECM remodeling, its overexpression degrades ECM and impairs keratinocyte migration [89].

ROS plays a dual role in tissue regeneration. At physiological levels, they are essential for angiogenesis, endothelial cell proliferation, and VEGF expression, supporting vessel formation and tissue repair [90]. In addition, ROS facilitates wound edge dilatation and stimulates the proliferation and migration of fibroblasts and keratinocytes, contributing to ECM formation and re-epithelialization [91]. However, excess ROS during wound healing is particularly harmful to regenerating tissues. On the one hand, excess ROS accelerates the senescence and apoptosis of fibroblasts, endothelial cells, keratinocytes, and mesenchymal stem cells, thereby significantly hindering the development of granulation tissue, angiogenesis, and epithelial cell proliferation [89, 92]. On the other hand, excess ROS can induce an increase in protease activity in skin tissue and accelerate collagen degradation in the ECM [93, 94]. For example, in chronic diabetic wounds, long-term hyperglycemia inhibits the migration and proliferation of keratinocytes, leading to insufficient re-epithelization of wounds and seriously affecting the wound healing process [95, 96]. Similarly, an overactive inflammatory response driven by neutrophils and the dysregulated expression of MMPs, such as MMP-9, contributes to ECM degradation and disrupts keratinocyte migration. These events collectively create a hostile wound environment that hinders progression through the diabetic wound healing phase [97].

The transition from skin injury to wound healing involves a complex series of changes within the wound microenvironment, where redox imbalance plays a critical role in disrupting inflammation, skin cells, and cytokine activity. The proliferation and remodeling processes are precisely regulated but remain vulnerable to disruption, particularly in chronic wounds. These pathological conditions highlight how imbalances in inflammatory responses, oxidative stress, and cellular dynamics can disrupt the healing process. Understanding the mechanisms behind chronic wound failure provides valuable insights for developing targeted therapeutic strategies. Approaches aimed at restoring redox homeostasis, modulating inflammation, and promoting cell proliferation and migration are key to improving chronic wound outcomes.

## The design and synthesis of nanozyme-driven multifunctional wound dressings

The microenvironment of chronic wounds is characterized by hypoxia, inflammation, and oxidative stress, with complications such as diabetes exacerbating hyperglycemia. These conditions impede angiogenesis and disrupt cellular function, complicating the healing process amid persistent inflammation and oxidative stress. Owing to their special physicochemical properties, nanomaterials can be tailored at the molecular level for drug delivery or can reasonably mimic the morphology and function of cells and tissues for tissue regeneration [98, 99].

**Fig. 2:**
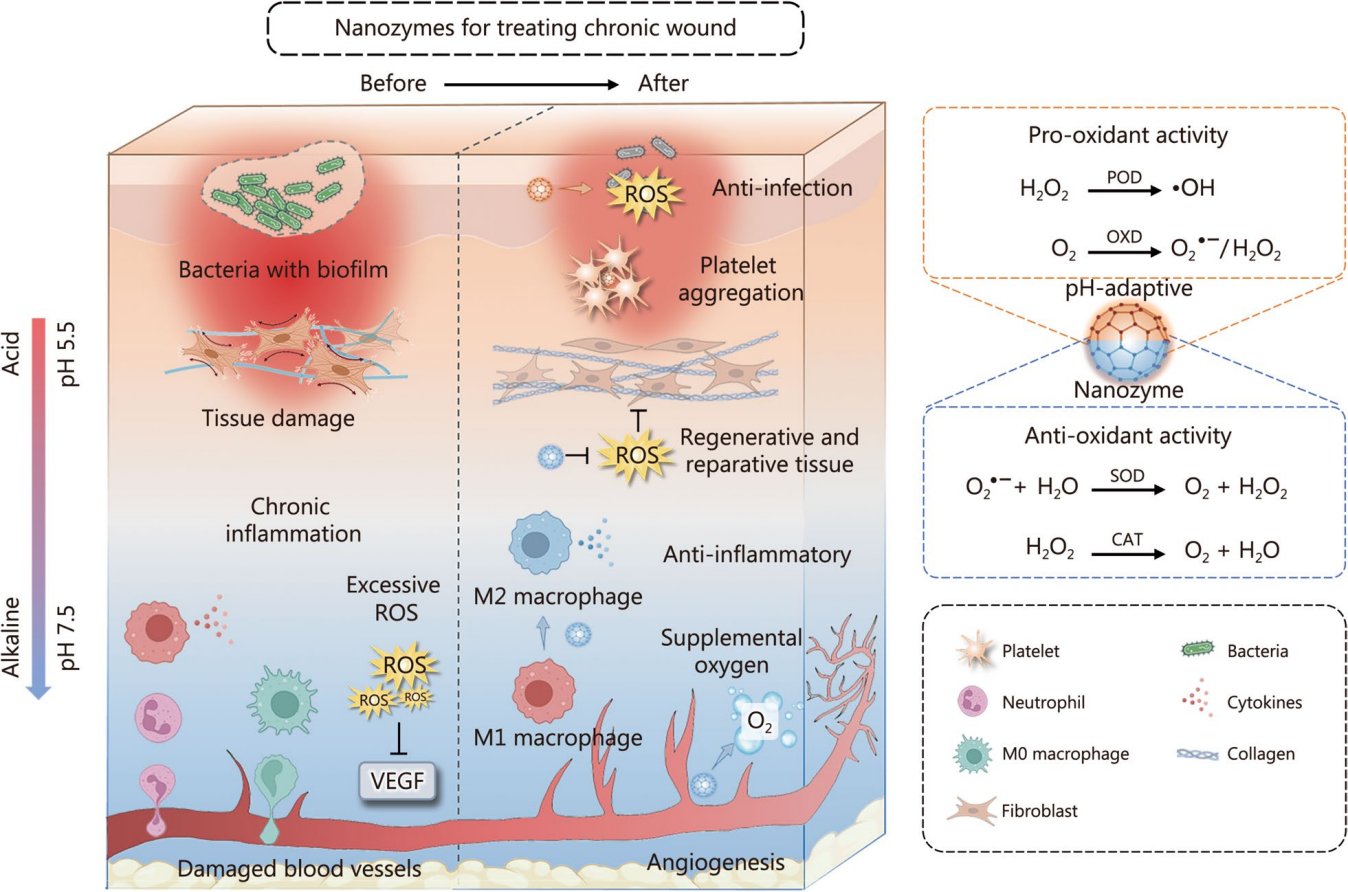
Overview of nanozyme-based therapeutic mechanisms for wound healing. Before treatment, pathogenic infections activate immune cells to infiltrate the wound site and release abundant antibacterial substances, but the biofilm formed by bacterial accumulation protects the bacteria, resulting in persistent inflammation. Furthermore, oxidative stress impedes angiogenesis and damages dermal cells, complicating tissue regeneration and prolonging the healing process. In contrast, nanozymes have the potential to modify the wound microenvironment. Their enzyme-like activity adapts to pH changes: under acidic conditions, they increase pro-oxidant activity to combat infection, whereas in alkaline environments, they shift toward anti-oxidant activity, reducing oxidative stress and inflammation. This dual functionality fosters a more conducive healing environment and promotes tissue regeneration. ROS reactive oxygen species, VEGF vascular endothelial growth factor, POD peroxidase, OXD oxidase, SOD superoxide dismutase, CAT catalase

As biocatalytic artificial enzymes, nanozymes are promising tools for modulating the dynamic balance of redox and abnormal biochemical environments in the wound microenvironment. Nanozymes can adapt their anti- or pro-oxidant capabilities in response to pH changes in the wound microenvironment, playing a vital role in breaking down harmful molecules and regulating redox balance and inflammation. In detail, oxidative stress can inhibit the anti-oxidant response and trigger the expression of inflammatory cytokines [100]. Nanozymes, such as Ce-based nanozymes, can counteract this by promoting the expression of the anti-inflammatory cytokine IL-10, thereby enhancing endogenous anti-oxidant defense [101]. Moreover, in autophagy regulation, nanozymes can upregulate autophagy-related genes (e.g., *BECN1*) while downregulating p62/SQSTM1, facilitating normal autophagic processes and mitigating inflammatory damage [102]. Nanozymes can also participate in regulating cell metabolism to improve chronic wound complications such as diabetes, remaining a major part in modulating glucose uptake, promoting glycogen synthesis, increasing insulin sensitivity, and reducing insulin resistance [103]. By improving the wound environment, nanozymes facilitate ECM reconstruction, a crucial step in tissue repair. These functions aid in the removal of detrimental substances and promote tissue regeneration (Fig. 2).

Despite their potential, the complex wound microenvironment poses challenges to the efficacy of nanozymes. Recent advances in nanozyme technology have spurred growing interest in their applications in regenerative medicine. As a novel class of wound dressings for clinical use, nanozyme-based dressings should carefully consider the choice of substrate and be designed to maximize therapeutic outcomes. This section explores common substrates and synthesis methods for nanozyme-based wound dressings, delves into their design principles, and offers insights to guide the development of next-generation dressings aiming to improve the treatment of chronic wounds.

### Selection of dressing matrix

The history of the wound dressing originated at approximately 2500 B.C. Egyptians first wrapped the wound with gauze and used honey as a wound healing accelerator. Owing to their anti-bacterial properties, ancient Greeks cleaned wounds with wine or vinegar [104]. Subsequently, passive auxiliary materials such as bandages, which may cause secondary damage when they are changed, provide limited protection for the wound [105]. Over the past few decades, dressings have become increasingly diverse, aiming to resist bacterial infection, increase oxygen tension, and provide a moist wound environment [106, 107]. Particularly, in recent years, a variety of smart wound dressings capable of continuously monitoring wound conditions in real-time with remarkable precision have been developed [108]. Smart wound dressings in the formats of biopolymers [109], hydrogels [110], foams [111], electrospun nanofibers [112], or composites have been integrated with drug release systems for effective wound management.

#### Biopolymer dressings

One of the conditions for the formation of an ideal wound dressing material is its mechanical properties, which are similar to those of human skin [113]. Natural materials like collagen, hyaluronic acid, chitosan, alginate, and elastin, whether derived from natural sources or synthesized artificially, exhibit excellent biocompatibility, biodegradability, nontoxicity, and adjustable permeability [114]. However, these materials often suffer from low stability, leading to increased storage and transportation costs [115]. To address this, biopolymer dressings are typically modified by blending with other materials to improve their stability.

#### Hydrogel dressings

Hydrogels are frequently used wound dressing that have good hydrophilicity, good biocompatibility, and a three-dimensional (3D) porous structure similar to that of the ECM. The exceptional mechanical properties endow hydrogels with perfect attachment to the wound site [116]. Moreover, these materials can be effectively applied and removed without secondary damage to the wound site with proper adhesion, resulting in a good healing experience for patients [117]. In addition to natural hydrophilic polymers such as chitosan, gelatin, hyaluronic acid, alginate, and dextran, synthetic polymers such as poly(ethylene glycol), poly(vinyl alcohol), and polyacrylamide have been employed in the fabrication of hydrogels via dynamic chemical or physical crosslinking [118]. The Schiff base reaction, a key player in click chemistry, has emerged as a vital technique for creating self-healing hydrogels, enabling the restoration of their structure and function after damage [119]. Initially, hydrogels often play simple roles in protecting wounds from the external environment and maintaining a moist internal environment. Owing to the multifunctional chemical or physical properties of the composition materials of hydrogels, they can be modified easily by bioactive substances or advanced sensors to achieve a variety of application functions, especially in antibacterial, anti-oxidant, and anti-inflammatory wound treatment and monitoring, which makes them competitive choices as wound dressing substrates [120, 121]. However, one of the disadvantages of these dressings is the inability to heal the wound with too much secretion [122].

#### Foam dressings

Foam dressings are distinguished by their porous structure, featuring many pores embedded within a solid polymer matrix, which imparts several desirable characteristics in wound healing. Specifically, they are lightweight, comfortable to wear, and easy to cut or shape without shedding fibers or particles [123]. Compared with hydrogels, they have excellent hemostatic performance and a strong ability to adsorb exudates. The role of absorbent dressings as either primary or secondary dressings is largely dependent on the exudate level, wound characteristics, and location [124], which are not applicable for dry or low-exudative wounds. Foam dressings are often combined with negative pressure wound therapy to remove thick exudate, facilitating wound management before and after surgical debridement [125]. At present, foam dressings composed of various materials, such as silicone-adhesive polyurethane [126], silicone [127], and alginate [128], have demonstrated great potential in the treatment of pressure ulcers [129]. To address the diverse requirements of healing wounds, foam dressings have undergone continuous innovation over the past few decades. For example, when it comes to edge design, highly adhesive silicone borders provide enhanced adhesion strength, effectively protecting wounds with a reliable barrier. In contrast, non-adhesive silicone edges are designed to minimize trauma to both the wound and the surrounding tissue during dressing changes [130].

#### Electrospun nanofibrous dressings

Electrostatic spinning is a technique in which polymer droplets overcome the surface tension barrier due to a high-voltage electrostatic field, resulting in the formation of a jet stream. This jet is subsequently elongated and solidified during the spinning process before being deposited onto a receiving substrate, effectively facilitating the direct production of polymer nanofibers [131]. Theoretically, any soluble or molten polymer material, including natural or synthetic polymers, can be electrospun [132]. Progressive morphological characteristics increase the porosity and oxygen permeability of electrospun nanofibrous wound dressings, which are beneficial for cell proliferation and tissue regeneration [133]. During electrospinning, these nanofibrous wound dressings are capable of efficiently incorporating various healing agents to achieve functional enhancement. However, owing to the material’s poor wettability, further crosslinking is needed to improve its properties. For example, polyvinyl alcohol (PVA), a synthetic polymer for developing electrospun nanofibrous wound dressings by electrospinning technology, is blended with other hydrophilic materials to improve hydrophilicity and adapt to the characteristics of wound dressings [134]. Moreover, the thinness of single-layer electrospun nanofiber membranes, coupled with their limited capacity to absorb exudate, can result in maceration and increase the risk of infection. To address this, hydrogel-attached or multilayer electrospun nanofiber membranes have been developed to enhance the water absorption properties of dressings [135]. Notably, Shi et al. [136] innovatively designed a dressing by electrospinning a hydrophobic polyurethane nanofiber array onto a hydrophilic microfiber network. This configuration enables the unidirectional drainage of excess biological fluids from the hydrophobic surface to the hydrophilic side of the wound, effectively managing the moisture balance.

### Strategies for preparing nanozyme-based wound dressings

Smart wound dressings that can deliver bioactive compounds such as nanozymes directly to the site of injury are introduced in Table 2 [137, 138]. This section will summarize in detail the strategies for preparing nanozyme-based wound dressings, which mainly include physical entrapment, chemical crosslinking, electrospinning, and self-assembly (Table 3) [139–163].

**Table 2:** Advantages and disadvantages of various wound dressings

Dressing types	Materials	Advantages	Disadvantages
Biopolymer dressings	Collagen, hyaluronic acid, chitosan, alginate, and elastin	Mechanical properties similar to human skin, biocompatibility, biodegradability, nontoxicity, and tunable permeability	Low stability, high storage, and transportation costs
Hydrogel dressings	Natural hydrophilic polymers and synthetic polymers	Good hydrophilicity, biocompatibility, 3D porous structure similar to ECM, excellent mechanical properties, no secondary damage to the wound site when removed, and appropriate adhesion	Limited effectiveness when secretions are excessive
Foam dressings	Silicone bonding polyurethanes, silicones, and alginates	Lightweight, comfortable to wear, easy to cut or shape, no shedding of fibers or particles, excellent hemostatic properties, and strong exudate absorption capacity	Not suitable for dry or low-exuding wounds
Electrospun nanofibrous dressings	Any soluble or fusible polymer material	Good porosity and oxygen permeability, easy to combine with various healing agents	Poor wettability

*3D* three-dimensional, *ECM* extracellular matrix

**Table 3:** Preparation methods for nanozyme-based wound dressings

Preparation strategies	Substrates	Featured nanozymes	References
Physical entrapment	Hydrogel	Ni_4_Cu_2_ hollow	[139]
	Hydrogel	MOF-818	[140]
	Hydrogel	Au/Cu_1.6_O/P-C_3_N_5_	[141]
	Hydrogel	Au-Pt alloy	[142]
	Hydrogel	Fe_3_O_4_@IHPs	[143]
	PVA	Fe_2_C/GOx	[144]
	Ni foam	Cr/CeO_2_	[145]
	Hydrogel	Cu_5.4_O@Hep-PEG	[146]
	Cryogel	MoS_2_@PDA	[147]
	Hydrogel	MnCoO@PLE/HA	[148]
	Hydrogel	MoS_2_-PDA	[149]
	Selenium-containing hybrid hydrogel	PB	[150]
	Hydrogel	WS_2_-CL	[151]
	Cryogel	Fe-MIL-88NH_2_	[152]
	Hydrogel	Curcumin-Zn-MOF	[153]
Chemical crosslinking	PVA	Hematite	[154]
	Hydrogel	MoS_2_	[155]
	Cryogel	MoS_2_	[147]
	Hydrogel	MnO_2_	[156]
Electrospinning	Polycaprolactone/gelatin/D-glucose composite fiber mesh	Fe-MOF	[157]
	Heparin sodium nanofibrous	PBNCs	[158]
	PVA/chitosan polymer	CeNPs	[159]
	PVA	PB	[160]
Self-assembly	Hydrogel	AgNPs@PDA	[161]
	Bacterial cellulose-enveloped polypropylene composites	Fe@HCMS	[162]
	Hydrogel	MnCoO@PDA	[163]

*MOF* metal-organic framework*, IHPs* indocyanine green (ICG) co-loaded hybrid nanoparticle, *PVA* polyvinyl alcohol, GOx glucose oxidase, *Hep-PEG* heparin-polyethylene, *PDA* polydopamine, *PLE/HA* polylysine/hyaluronic acid, *PB* prussian blue, *CL* catechol, *MIL* Matériaux de l’Institut Lavoisier, *NPs* nanoparticles, *NCs* nanocrystals, *HCMS* hollow mesoporous nanocatalyst

#### Physical entrapment and chemical crosslinking

Physical entrapment is often used to entrap metallic nanozymes of ultrasmall sizes into dressing materials, especially hydrogels, owing to their porous structures, which mimic the human ECM environment and are easily loaded with therapeutic agents. The composition can be made directly by mixing or soaking hydrogels with powdered nanozymes [139, 146] or by blending nanozyme solutions with hydrophilic polymers capable of forming hydrogels through chemical reactions [142].

However, physically entrapped nanozyme-based dressings may have many disadvantages, such as uneven mixing and unresponsive release. Chemical crosslinking is another commonly used measure to graft nanozymes into dressings. The addition of crosslinking agents can enhance the mechanical strength and tissue bonding attributes of dressings. Acryloyl PF127 (PF127-DA), obtained from the reaction of PF127 with triethylamine dissolved in anhydrous dichloromethane, is extensively used as a crosslinking agent in the fabrication of wound dressings [149]. Li et al. [152] designed metal-organic frameworks nanozyme composite cryogels by grafting Fe-MIL-88NH_2_ nanozymes to glycidyl methacrylate-functionalized dialdehyde chitosan (dialdehyde GMA-CS) via a Schiff base reaction and using PF127-DA as a crosslinking agent, ultimately reaching a pH-responsive nanozyme release therapeutic effect. Based on the chemical reactions between different groups, the addition of various noncovalent interactions (such as hydrogen bonds, hydrophobic interactions, and electrostatic interactions) can endow dressings with good tensile properties. Xie et al*.* [151] proposed a dual-crosslinking strategy involving physical hydrogen bonds to prepare bioadhesive hydrogels, which are highly stable.

#### Electrospinning

Electrospinning allows homogeneous integration of nanozymes in the resulting fibers [154]. In addition, researchers can conveniently realize the quantitative assembly of nanozymes by adopting different measures to load graded amounts of nanozymes onto them. Oh et al*.* [160] prepared electrospun PVA nanofibers by incorporating chitosan-stabilized Prussian blue nanoparticles (NPs) at an optimized weight ratio to select the best product with the ability to scavenge ROS. Zhang et al*.* [157] developed a polycaprolactone/gelatin/D-glucose fiber mesh by depositing various amounts of glucose oxidase (GOx)-Fe-metal–organic framework (G@Fe) inside the pores and on the surface of the fiber mesh via an electrospinning process to enhance wound healing.

#### Self-assembly

Self-assembly is the process of individual units of a material associating into highly ordered structures/patterns via noncovalent interactions, imparting unique properties to both inorganic and organic structures [164]. In recent years, nanozyme-based dressings inspired by mussels have attracted widespread interest. Mussels show strong adhesiveness and self-setting ability because the catechol side chain of 3,4-dihydroxyphenylalanine contained in mussels reacts with various substrate surfaces [165]. Jia et al*.* [166] developed a new route for producing adhesive hydrogel bioelectronics, which can undergo self-setting by triggering free-radical polymerization with ultrasmall tannic acid-chelated Ag nanozyme catalysis. In this way, nanozymes can not only be incorporated into hydrogels but also promote the self-assembly of hydrogels to achieve dressing preparation.

### Design of nanozyme-based wound dressings

The treatment approach that relies solely on enzyme-like activity is limited, as it fails to comprehensively address the multifaceted challenges of oxidative stress, infection, and chronic inflammation present in traumatic wounds. In contrast, nanozyme-driven dressings can significantly alter the wound microenvironment and enhance therapeutic outcomes through their unique functional synergies, including their anti-oxidant properties, antibacterial activity, angiogenesis-promoting effect, and immunomodulatory capacity. In this section, we delineated the design strategies for multifunctional nanozyme wound dressings, intending to explore how to augment the efficiency and efficacy of wound healing by integrating these multifunctional nanozyme-driven dressings. This analysis offers valuable strategies and insights for the future clinical deployment of nanozyme wound dressings.

#### Responsive design

When designing a nanozyme-based wound dressing, it is essential to prioritize its environmental responsiveness. An ideal responsive wound dressing should exhibit intelligent reactivity and be able to respond to fluctuating conditions within the wound microenvironment, such as variations in pH levels, redox states, and biochemical molecules. This adaptability ensures the controlled and appropriate release of nanozymes, optimizing the healing process.

Persistent inflammation, bacterial infection, and other biochemical factors result in a fluctuating pH microenvironment in chronic wounds. Under these conditions, researchers have skillfully used the pH-dependent enzyme-like activity of nanozymes to play a catalytic role in different stages of wound treatment. The copper hydrogen phosphate (CuP) nanozyme dressing based on an alginate hydrogel designed by Feng et al*.* [167] has pH-responsive peroxidase/catalase (POD/CAT)-simulated catalytic activity. In infected wounds (low pH environment), the dressing has a strong bacterial eradication effect, and in chronic diabetic wounds (high pH environment), it has a remarkable angiogenic effect. However, relying solely on the enzyme-like activity of nanozymes is insufficient to address the dynamic changes in this microenvironment. Nanozyme-based wound dressings should also provide a platform for the controlled release of nanozymes to improve their catalytic activity. In nanozyme-based wound dressings synthesized by chemical crosslinking, this controlled release can be realized by breaking chemical bonds. For example, Schiff base bonds are reversible under acidic conditions, allowing them to break and reform at various pH values, which enables nanozymes to be released in acidic environments and re-crosslinked with materials when the pH changes [152]. This design allows intelligent adaptation of nanozyme-based wound dressings to varying conditions of the wound microenvironment, not only enabling the flexible and regulated release of nanozymes but also reducing the excessive accumulation of nanozymes, thereby increasing their biological safety.

In addition to pH responsiveness, the potential of other wound microenvironmental factors can also be harnessed to trigger nanozyme release. High levels of ROS and MMPs are present in the microenvironment of chronic persistent wound inflammation. Xu et al*.* [168] combined the copper tannic acid coordination nanosheets (CuTA NSs) and the triglycerol monostearate/2,6-di-tert-butyl-4-methylphenol (TM/BHT) hydrogel to form TM/BHT/CuTA hydrogel system. CuTA nanozymes can be released in response to increased MMP and ROS levels in the inflammatory environment and exhibit CAT-like and superoxide dismutase (SOD)-like activities to scavenge ROS. Capitalizing on the elevated glucose levels characteristic of diabetic wounds, Liang et al*.* [169] developed a pH/glucose dual-responsive hydrogel for dissociation that facilitates nanozyme release. This was achieved through the incorporation of two dynamic covalent bonds, a Schiff base and phenylboronic acid, highlighting their versatility in responsive material design. The latter, through its interaction with glucose, triggers the dissociation of phenylboronic acid and catechol moieties, thereby increasing the release of nanozymes. Inspired by bacterial enzymes that destabilize host tissue cells to initiate infection, the macromolecular dressing matrix is designed to respond to relevant enzymes, enabling intelligent release of bactericidal agents for targeted antimicrobial action. Tian et al*.* [170] developed nanozyme-based hydrogels by combining Fe with hyaluronic acid through chemical crosslinking, which could achieve enzyme-responsive controlled release. Triggered by hyaluronidase from nearby bacteria, this hydrogel could locally disintegrate and release Fe^3+^ for bacterial eradication through the Fenton reaction, demonstrating significant antibacterial effectiveness across various strains. Moreover, owing to the interaction between Fe^3+^ and COOH ligands, this hydrogel can autonomously repair in minutes, indicating promising prospects for bioengineering applications on exposed biological surfaces.

Currently, the design of responsive nanozyme-based wound dressings focuses significantly on real-time surveillance of the wound microenvironment. This allows for immediate and targeted interventions, which are crucial to expedite the recovery of chronic wounds. Wang et al*.* [171] loaded carbon quantum dots with cerium oxide-molybdenum disulfide NPs (C@M@P) along a polydopamine (PDA) layer into a hydrogel, resulting in the preparation of a diagnostic and therapeutic hydrogel (LAMC/CD-C@M@P). The doping of carbon quantum dots enabled the hydrogel to exhibit fluorescent changes in response to pH under ultraviolet light, which allowed the use of a smartphone to collect images of the hydrogel and convert them to wound pH signals, providing a means for the early detection of bacterial infections. This multifunctional wound dressing enables the combination of diagnostic and treatment and represents a significant advancement in chronic wound management.

#### Optimal design

Strategically engineered nanozymes can enhance their enzyme-like activities, yet their standalone use in clinical settings is limited by the multitude of factors that can impede their efficacy. The intelligent design of nanozyme-based wound dressings can complement the catalytic action of nanozymes, leading to more efficient modulation of the chronic wound microenvironment and thereby accelerating wound healing. A common strategy involves incorporating natural enzymes into nanozyme wound dressings, which helps trigger cascade catalytic reactions, significantly boosting the therapeutic effects of nanozyme-based dressings. For example, GOx is frequently used in managing diabetic wounds due to its ability to consume glucose and produce H_2_O_2_, a key substrate for nanozymes. This process enhances therapeutic outcomes, promoting sustained production of ROS and O_2_, which are antibacterial and relieve hypoxia [172, 173].

As research on nanozymes has shifted its focus from serendipitous discovery to a more systematic approach, investigating electron transfer mechanisms provides deeper insights into the catalytic processes of nanozymes [174]. This understanding is crucial for the enhancement of their enzyme-like activity, enabling their effective application in chronic wound treatment. Ligand–metal charge transfer was induced by Chen et al. [17] by coordinating polyphenol ligands on the Cu_3_(PO_4_)_2_ NSs, which led to a strong charge transfer and regulated the valence states of copper. Eventually, this modification significantly improved the ROS-scavenging ability of the nanozyme in wound healing. On the other hand, Li et al. [175] focused on bioheterojunction engineering derived from a metal–organic framework with a Cu–O–Zn bond at the interface. This bioheterojunction facilitated electron redistribution, leading to ultrahigh CAT-like activity and sustained ROS consumption with enhanced biocompatibility and cell adhesion, further promoting wound healing. Emphasizing the catalytic mechanisms of nanozymes and focusing on engineered design strategies are able to minimize the redundant modifications in nanozyme-based wound dressings. In this way, unforeseen biological effects from the integration of disparate materials can be prevented.

Photothermal therapy (PTT), which leverages the photothermal effect of photothermal agents, is a novel combined therapy for wound healing. By inducing localized hyperthermia, PTT enhances blood circulation, stimulates the proliferation and differentiation of cells surrounding the wound, and promotes tissue regeneration and reconstruction. This focused, therapeutic approach hastens the wound healing process, positioning it as a promising treatment option for various wound types [176]. PTT exhibits considerable promise in wound recovery, particularly in its antibacterial capabilities. However, high temperatures can cause permanent harm to healthy tissue. Such damage hinders the processes of collagen formation and blood vessel growth, which negatively impacts the tissue’s overall healing. Li et al. [177] developed a Cu_2_O-SnO_2_-PDA heterojunction nanozyme-doped hydrogel, where the incorporation of PDA endowed the dressing with superior photothermal conversion capabilities. This enhancement enabled the dressing to perform an outstanding synergistic therapeutic role, combining chemodynamic therapy and PTT at moderate temperatures. This design demonstrates that nanozyme-driven dressings can enhance the efficacy of combined therapies, boost nanozyme catalytic activity, and better protect the wound environment.

Employing multiple strategies to enhance the catalytic activity of nanozymes is a common and effective approach to optimizing wound dressing designs, rooted in the principles of nanozyme-based catalytic therapy. Nonetheless, enhancing the catalytic activity of nanozymes does not always lead to improved therapeutic outcomes. Future research should prioritize establishing a clear and direct correlation between the design of nanozyme-based wound dressings and their therapeutic efficacy rather than focusing solely on increasing their catalytic performance.

#### Multifunctional design

The microenvironment of chronic wounds is characterized predominantly by hypoxia, inflammation, and oxidative stress, which impedes angiogenesis, disrupts cellular functions, and decelerates the healing process due to persistent inflammation and an imbalanced redox state. This intricate wound microenvironment poses significant challenges to the practical clinical application of nanozyme catalytic therapy. Consequently, enhancing the catalytic activity of nanozymes alone is insufficient. Nanozyme-driven dressings offer a multifunctional platform that enables the design of a more targeted treatment strategy tailored to the dynamic changes in the overall wound microenvironment, leveraging the activity of nanozymes. This approach ensures a comprehensive and adaptive therapeutic intervention for chronic wounds.

ROS is a double-edged sword in the process of wound healing [178]. Nanozymes with POD-like activity can convert H_2_O_2_ into hydroxyl radicals (·OH) in an acidic environment, thus achieving sterilization, but they may also lead to ROS-mediated inflammation [179]. Nanozyme-based wound dressings can be combined with natural antibacterial agents to alleviate this contradiction by assisting in nanozyme catalytic therapy. Proanthocyanidins, a class of natural polyphenols derived from plants, play multifaceted roles as anti-oxidants, antimicrobial, and anti-inflammatory agents. This integration helps reduce the possibility of excessive ROS-induced inflammation by balancing the production of ROS, thereby enhancing the ability of dressings to provide comprehensive protection and promote wound healing [180]. Similarly, the natural polyphenol chlorogenic acid in a nanozyme wound dressing designed by Wei et al. [181] as a mild antibacterial agent, assisted by the CAT-like activity of the nanozymes, can effectively inhibit wound infection, alleviate wound hypoxia, and inhibit oxidative stress.

ROS are challenging to eliminate in the chronic wound microenvironment, as they trigger pathological signaling, recruiting immune cells that overproduce inflammatory cytokines (including ROS), creating a ROS-inflammation vicious cycle that impairs healing. The key to breaking this cycle is to remove ROS and capture pro-inflammatory factors, prevent damage caused by excessive ROS downstream and upstream, and thus accelerate wound healing. While nanozymes mimicking anti-oxidant activities can reduce ROS levels, they often fail to fully resolve the underlying chronic inflammation. This dilemma can be solved by introducing bioactive substances, such as heparin, into the wound dressing and the mutual attraction between its sulfate group and the positively charged amino acid residue of inflammatory factors, thus capturing inflammatory factors [146, 158]. Combined with the anti-oxidant therapeutic activity of nanozymes, this multifunctional combination can prevent persistent inflammation in chronic wounds and promote the progression of wound healing.

In addition to regulating oxidative stress and the persistent inflammatory state of the wound microenvironment, nanozyme-driven wound dressings can be further designed to promote angiogenesis, which is also very important for the healing of chronic infection wounds. L-arginine, the donor of endogenous NO, has been widely proven to promote angiogenesis, thus regulating vasodilation and promoting angiogenesis [72], and is strategically designed for use in nanozyme-driven wound dressings. The produced NO can not only promote the formation of new blood vessels and improve microvascular blood flow but also react with ROS catalyzed by nanozymes to generate peroxynitrite, further eradicating bacterial biofilms [141, 147]. This multifunctional approach not only accelerates wound healing but also delivers potent antibacterial efficacy.

The essence of multifunctional design lies in addressing multiple challenges in the wound microenvironment, such as infection, inflammation, and oxidative stress, to facilitate comprehensive wound healing. However, overly complex designs may result in functional competition, potentially diminishing therapeutic efficacy. To mitigate this, future research should focus on understanding and optimizing the synergistic interactions between functions to avoid the pitfalls of “overdesign”. Moreover, the complex wound microenvironment underscores the demand for real-time monitoring to realize the integrated diagnosis and treatment process of nanozyme-based wound dressings from release and catalytic reactions to curative effects.

## Treatment with nanozyme-based wound dressings

Overall, various dressing materials can effectively function as carriers for nanozymes through physical or chemical interactions. Nanozyme-based wound dressings have been extensively developed for applications in infected wounds, diabetic wounds, and brain injuries [145, 182], primarily by restoring the normal redox environment and inflammatory homeostasis. In this section, we offer an overview of the latest research developments concerning nanozyme-based wound dressings for the treatment of infected wounds and diabetic wounds.

### Infected wounds

As the largest organ of the body, the main function of the skin is to isolate it from the outside world. Severe burns and infections caused during surgery significantly increase the mortality rate of patients [183, 184]. *Staphylococcus aureus*, *Pseudomonas aeruginosa*, and methicillin-resistant *Staphylococcus aureus* are common types of pathogenic microorganisms that infect wounds [185]. The traditional treatment for bacterial infection involves the use of intravenous drip/oral/topical antibiotics after debridement to achieve therapeutic effects. Penicillins, cephalosporins, chloramphenicol, tetracyclines, colicins, macrolides, and analogs of these antibiotics are commonly used [186]. However, the disaster of drug resistance caused by the abuse of antibiotics is still a crisis that cannot be ignored. Owing to both the discovery of new antibiotics and the chemical modification of existing drugs, their low target selectivity is a major problem in clinical treatment [187]. Nanozymes can be combined with other components (aptamers, peptides, nucleic acid probes, antibodies, and antibiotics) to achieve selective antibacterial effects via their nanomaterial characteristics. For example, polymyxin B can be immobilized on the surface of a nanozyme by electrostatic assembly, and targeted release can be achieved via microneedles [188]. This is highly beneficial for increasing drug accumulation, reducing overall drug exposure, and promoting bacterial absorption. Moreover, the rational design of new drugs based on novel antibacterial mechanisms, such as disrupting the redox balance of bacteria, maybe a crucial step in addressing antibiotic resistance and promoting wound healing. Nanozymes offer numerous distinct advantages in antimicrobial applications, particularly in the battle against antibiotic-resistant bacteria [189].

Notably, reducing glutathione (GSH) levels is also an effective approach to increase the degree of oxidative damage to bacteria caused by ROS. An intelligent multifunctional mixed nanozyme composed of horseradish POD modified with PDA NPs and ultrafine Fe_3_O_4_ NPs was constructed by Xiao et al*.* [190]. The therapeutic efficacy of chemodynamic therapy was enhanced by the simultaneous elimination of GSH and the in-situ generation of OH at the site of infection, facilitated by magnetically targeted, long-term antibacterial interventions. Sometimes, there is insufficient H_2_O_2_ in the microenvironment, resulting in an insufficient concentration of free radicals. Hence, nanozymes with various enzyme-like activities should be designed to promote the production of ROS. Nanozymes with POD- and oxidase-like activities were designed to further catalyze the formation of H_2_O_2_ from O_2_, which is further converted to ·OH [191]. Nonetheless, in cases of cutaneous injury, microvascular leakage leads to increased pH in the wound microenvironment, resulting in a mildly alkaline environment that promotes bacterial proliferation. Consequently, this hinders the optimal catalytic performance of most nanozymes in mimicking POD-like activity. In response to this contradiction, improving the POD-mimetic behavior of nanozymes in neutral or basic environments through various strategies, including photodynamic therapy and sonodynamic therapy, is essential [192].

In contrast to the ability of nanozymes to manage the intricate infectious wound milieu, enhancing the hydrolase activity of these nanozymes allows for a more direct and efficient bacteria-killing approach, thus offering a novel avenue for the advancement of antibacterial nanozyme-based wound dressings. Nuclease-mimicking nanozymes have the potential to remove extracellular DNA produced by bacteria, indicating the possible mechanisms and application potential of nanozymes for sterilization [193]. Xu et al. [194] proposed CeO_2_ NPs as deoxyribonuclease I-mimicking nanozymes that can tightly adsorb DNA and cleave it without obvious sequence preference in hydrolytic reactions instead of in redox reactions. Ce (III) and Ce (IV) act together, binding to the two nonbridging oxygen atoms of the phosphate group to be cleaved. A hydroxyl group on CeO_2_ acts as a nucleophile to attack the phosphorus atom and initiate the cleavage reaction. It implies that the treatment of infected wounds should evolve to incorporate strategies and designs that leverage simple antibacterial mechanisms tailored to the specific characteristics of pathogenic microorganisms’ life activities. Nevertheless, this strategy needs to consider whether the hydrolase activity of nanozymes affects normal cells, which requires more attention in the development of nanozymes with deoxyribonuclease I-like activity specific to the bacterial biofilm DNA sequence.

Recognizing the critical role of pH in the wound healing process, researchers have focused on the creation of diverse pH-sensitive wound dressings. The optimal bactericidal efficacy of nanozymes may be attained by focusing on the modulation of the pH of the wound microenvironment. Yuwen et al*.* [195] prepared electrospun nanofibers serving as carriers for ZnO_2_ NPs, Fe_3_O_4_ NPs, and polyacrylic acid (PPF/PZ nanofibers), in which polyacrylic acid can provide H^+^ to promote the production of H_2_O_2_, provide an acidic environment and sufficient substrate for the nanozymes to exert POD-like activity, and increase the production of ·OH to kill bacteria. To increase the antibacterial efficacy of nanozymes under nonacidic pH conditions, Fe_3_O_4_ NPs and CeO_2_ nanocrystals were functionalized with adenosine triphosphate to increase POD-like activity under physiological conditions by Vallabani et al. [196]*.* As a result, these pH-independent nanozymes demonstrated considerable antibacterial efficacy against both gram-positive and gram-negative bacterial strains under neutral pH conditions.

Some bioactive substances, such as chitosan, have certain antibacterial activities and have been used to form hydrogel wound dressings, which have attracted widespread attention because of their easy availability, low extraction cost, good biocompatibility, and biodegradability [197]. However, nanozyme-based wound dressings are significantly superior to those based on naturally active substances in terms of antibacterial performance, versatility, intelligent response, and complex wound treatment. The stability and durability of nanozyme-based wound dressings in complex environments offer diverse options for chronic wound treatment.

### Diabetic wounds

Diabetes mellitus is a metabolic disease characterized by hyperglycemia that can be divided into two types. Insufficient insulin secretion or insulin insensitivity eventually causes diabetes mellitus [198]. In addition to the harmful effects of hyperglycemia on their metabolic system, diabetic wounds, which are among the most persistent complications of diabetes, are difficult to heal [64]. The types of diabetic wounds usually include ischemic wounds, infected wounds, tension wounds, diabetic foot ulcers (DFUs), and bedsores. The common feature of these wounds is vasculopathy due to blood glucose abnormalities, resulting in an ischemic and hypoxic tissue environment accompanied by severe bacterial infection. Consequently, the delayed healing of diabetic wounds is associated with a cascade of alterations induced by hyperglycemia, including shifts in the wound microenvironment, perturbations in acid–base equilibrium, and diminished cellular immunity and resistance [199, 200]. Injecting insulin or taking hypoglycemic drugs, the general method for controlling blood glucose levels in diabetic patients, has several side effects, such as edema, hypoglycemia, obesity, gastroparesis, and skin allergies [201, 202]. Apart from antibiotics, current clinical approaches for managing diabetic wounds include surgical debridement, various dressings, and hyperbaric O_2_ therapy [203]. Nevertheless, these methods are inadequate because of their shortcomings, such as high cost, inconvenient treatment, and ignorance of the microenvironment in diabetic wounds. By delivering localized and precise action, nanozymes minimize systemic side effects, while their integrated intelligent response features dynamically adjust therapeutic effects based on the wound state, significantly increasing treatment accuracy and sustainability [204], which makes up for the shortcomings of traditional methods.

Hyperglycemia in diabetic patients can cause lesions in the vasculature, leading to hypoxia and interference with the oxidation of glucose to produce free radicals, which can cause oxidative stress and damage tissues and cells [205]. Overexpressed ROS are also responsible for inducing inflammatory cells and MMP overactivation [206]. In addition, the hyperglycemic wound environment is undoubtedly a suitable petri dish for sustained bacterial growth. Therefore, to accelerate wound healing in patients with diabetes, controlling the blood glucose level is a key objective. GOx can catalyze the conversion of glucose into gluconic acid, which can be considered a viable method for diabetic wound healing [172]. However, the use of natural GOx has the disadvantages of instability and high cost, so increasing the development of nanozymes with GOx activity is necessary. Subsequent studies revealed the GOx-like catalytic mechanism of Au NPs, wherein hydrated glucose anions bind to gold, enabling O_2_ to form an O–Au–O–O⁻ bridge via nucleophilic attack, thereby transferring electrons from glucose to O_2_ to generate gluconic acid and H₂O₂ [207–210]. Other noble metal nanomaterials, such as Ag, Pt, and Pd, have also been proven to have GOx-like activities because of their excellent electronic and optical properties [211, 212]. Zhang et al*.* [142] fabricated a versatile hydrogel bandage incorporating Au-Pt alloy NPs. By optimizing the Au-to-Pt ratio for diabetic wound care, the Au-Pt alloy NPs exhibit optimal GOx- and CAT-like activities, which can both break down H_2_O_2_ into O_2_ to alleviate hypoxia and metabolize glucose. Although the integration of natural enzymes into diabetic wound dressings is a common practice to increase the effectiveness of catalytic therapies and initiate cascade reactions, the high cost and limited stability of traditional enzymes, such as GOx, hinder their broad application. To address these issues, we can design nanozymes that mimic GOx activity, offering a cost-effective alternative that not only replaces natural enzymes but also enhances the functionality and reduces expenses through material optimization.

Alterations in the microenvironment due to hyperglycemia in diabetic individuals increase their risk of bacterial infections. Tu et al. [156] combined hyperbranched poly-L-lysine with MnO_2_ NSs through chemical crosslinking to create a novel multifunctional wound dressing that boasts enhanced dispersion stability and superior antibacterial capability. Unlike the previously discussed disinfection mechanism, this hydrogel dressing is capable of neutralizing various ROS species to produce O_2_ and stimulate NO synthesis, effectively eliminating methicillin-resistant *Staphylococcus aureus*. Compared to hydrogel dressings, microneedle dressings demonstrate superior antibacterial efficacy in diabetic wounds by combining precise penetration of thickened stratum corneum/biofilm with multilayer protective backing (e.g., chitosan/PVA) for enhanced targeted delivery and comprehensive protection [144, 213].

Based on the intricate nature of diabetic wounds, researchers have explored the cascade reactions of nanozymes possessing multiple enzyme-like functions to investigate the balance of the redox microenvironment in wounds [214–216]. However, the effectiveness of treatments has been restricted by the overlapping enzyme-like activities of nanozymes and the wound microenvironment. Further examination is necessary to understand the competitive mechanism among nanozymes with diverse enzyme-like properties for the development of wound microenvironment-responsive therapeutic solutions and the enhancement of wound treatment approaches. Shang et al. [141] formulated a nanozyme-enriched hydrogel spray comprising hyaluronic acid-encapsulated L-arginine, ultrasmall gold NPs, and Cu_1.6_O NPs along with phosphorus-doped graphitic carbon nitride (ACPCAH) NSs designed for the treatment of DFUs augmented with ultrasound therapy. The mechanism of the SOD-CAT-GOx-POD/nitric oxide synthase five-enzyme-like cascade reaction activated by the DFU microenvironment, including the release of hyaluronidase and pH changes, was meticulously studied. Both in vitro and in vivo experiments revealed the remarkable DFU therapeutic effects of ACPCAH, which include reducing inflammation, alleviating hypoxia, lowering blood glucose levels, enhancing angiogenesis, eliminating pathogenic bacteria, and promoting the healing of diabetic wounds. This study describes a wound microenvironment-responsive integrated diabetic wound treatment strategy utilizing multienzyme-like nanozymes. It also enhances the format of wound dressing sprays, showing potential for clinical applications.

## Potential safety issues and toxicity of nanozymes in long-term use

Nanozymes hold great potential as therapeutic agents for chronic wound dressings, primarily because of their unique catalytic properties and multifunctional design capabilities. They effectively regulate the microenvironment of chronic wounds by exhibiting antibacterial, anti-inflammatory, and anti-oxidant activities, thereby promoting wound healing. Compared with traditional treatments for chronic wounds, nanozymes offer several advantages, including enhanced stability, prolonged effectiveness, and versatile application. In addition, combined with intelligent response designs, such as pH- or ROS-triggered release systems, nanozyme dressings can also achieve targeted therapy and precise regulation, which provides new ideas for the personalized treatment of chronic wounds. While nanozyme-based wound dressings have demonstrated remarkable efficacy in accelerating chronic wound healing, their clinical translation, particularly for long-term use, necessitates a thorough understanding of potential safety concerns and in vivo toxicity. This section explores the biocompatibility of nanozymes, potential side effects, and currently approved nanomaterials, aiming to provide valuable insights for researchers in the field.

### Biocompatibility of nanozymes

Biocompatibility refers to the ability or tendency of a substance to cause adverse reactions in biological systems. The biocompatibility of nanozymes is influenced by many factors. First, the shape of the nanozyme has a significant influence on its biocompatibility. Studies have shown that spherical NPs are usually more biocompatible than nanoflowers are [217–219]. This is because the shape of the spherical particles has little influence on the cell membrane, which reduces the adverse effects on cell activity and metabolism. A greater surface area and edge characteristics, such as nanoflowers or NSs, may lead to cell membrane damage, thus causing biocompatibility problems. The size of nanomaterials also influences their biocompatibility [220]. Nanozymes with smaller particle sizes have greater permeability and specific surface areas, which is beneficial for the treatment of deep wounds, but they may face problems of rapid clearance and reunion. Larger particles have longer retention times and greater stability, which is suitable for long-term treatment of superficial wounds, but their permeability is poor, which may increase local inflammatory reactions. Therefore, the particle size of nanozymes for treating chronic wounds needs to be adjusted according to the required wound types and the functional requirements of the nanozymes to balance permeability, catalytic activity, biocompatibility, and metabolic clearance.

In the treatment of chronic wounds, surface modifications of nanozymes are widely employed to enhance their properties, with polymer encapsulation being the most common approach, utilizing materials like polyethylene glycol [150], PDA [163], ε-polylysine [221], and chitosan [222]. These methods increase the stability of nanozymes and minimize immune responses. Peptide and glycosylation surface modifications improve the affinity of nanozymes for target cells and reduce damage to normal tissues [223]. The use of biodegradable materials in nanozyme construction not only reduces in vivo toxicity but also improves treatment safety [224]. Additionally, combining nanozymes with bioactive substances amplifies their therapeutic efficacy and further enhances their biocompatibility [225, 226]. Through these modification strategies, nanozymes can offer safer and more effective treatments in chronic wound care.

Although there are many ways to improve the biocompatibility of nanozymes, it is necessary to understand the complex interactions between NPs and biological systems, which is very important for designing nanomaterials with low inflammation, targeted organ delivery, and high biocompatibility. Dong et al. [227] proposed an interpretable causal system optimization framework to improve the biocompatibility of nanomaterials through machine learning and optimization algorithms. The interpretable causal system optimization framework integrates upstream machine learning prediction and downstream nanoparticle optimization tasks, screens out the key factors that affect the immune response and organ burden, and quantifies the threshold of the biological response, providing quantitative information and constraints for designing nanomaterials with high biocompatibility and targeted organ delivery. Machine learning can provide quantitative information and constraints for the design of nanomaterials so that researchers can better understand the complex relationships between the properties of nanomaterials and their biological effects.

### Potential side effects of nanozymes

Compared with natural active substances, antibacterial agents, and natural enzymes, one of the major problems with nanozymes is their toxic side effects, which is a major challenge for their clinical biomedical application. Determining the cell and organ toxicity of nanozymes is indispensable in biomedical application research. The direct contact of nanozymes at the wound site may cause toxicity to local cells (such as keratinocytes, fibroblasts, and immune cells). For example, at a certain concentration, iron oxide nanozymes may inhibit cell proliferation and destroy membrane integrity by overproducing ROS or releasing metal ions [228]. Although manganese dioxide nanozymes have anti-oxidant effects, they may inhibit the phagocytosis of macrophages at high concentrations, resulting in an abnormal local immune response [229]. In addition, nanozymes may penetrate or be absorbed into the blood through the wound, accumulate in metabolic organs, and cause potential organ toxicity.

The methods used to improve the toxicity of nanozymes mainly focus on biocompatibility, dosage control, and individualized design to ensure their curative effect and reduce potential damage to normal tissues and organs. The dosage of nanozymes is a critical factor in toxicity control. Typically, this is managed by adjusting the amount of nanozymes applied per square centimeter of the wound, ensuring that the effective dose for bacteriostasis and antioxidation remains safe below the threshold for cytotoxicity or systemic toxicity. By incorporating local responsive designs (such as pH, ROS, and temperature sensitivity), the release of nanozymes can be precisely controlled. Additionally, the delivery mode of nanozymes can be optimized for accurate dose regulation. For example, microneedle delivery ensures a uniform distribution of nanozymes, allowing each microneedle to deliver a consistent dose [230]. The length, diameter, and load of the microneedles can be adjusted to control the dosage and range of nanozymes delivered. Furthermore, dosage control can be tailored to different wound treatment needs through sustained release strategies [231]. Nevertheless, NPs of different sizes are enriched in different organs. In general, nanomaterials smaller than 20 nm tend to accumulate in the kidney, whereas nanomaterials 20 − 100 nm in size are preferentially deposited in the liver [232]. This organ enrichment and organ toxicity also change according to the material and dosage of the nanozymes. In practical applications, personalized nanozyme materials are tailored according to the patient’s health status, liver and kidney function, and immune response. The optimal size and dosage are determined through a comprehensive evaluation of the patient’s physiological conditions. In the future, it is essential to systematically assess the in vivo distribution and metabolic efficiency of nanozymes in animal models, explore safe dose ranges for different types of nanozymes, and consider the potential long-term toxicity risks of material degradation products. In conclusion, while nanozymes show promising potential as wound dressings for short-term applications, their safety must be further clarified and optimized through continued research to ensure reliable and effective use in clinical settings.

### Approved nanomaterials with potential for wound healing

In recent years, with increasing research on nanomaterials, significant attention has focused on their biomedical applications. In the field of wound treatment, silver NPs, which have been approved for commercialization, have been incorporated into wound dressings to treat infectious wounds. Similarly, feraheme, a drug approved for treating iron deficiency in chronic kidney disease, is essentially composed of iron oxide NPs. Iron oxide NPs have also demonstrated antibacterial properties in various studies, indicating their potential as a wound dressing agent [233–235]. Another notable development is the use of Nanobiotix-3, a functional HfO_2_ NPs approved by the European Union as a radiosensitizer, which holds promise for exploring enzyme-like activity and could be applied in catalytic wound therapy. Furthermore, the PB nanodrug Radiogardase has gained Food and Drug Administration approval. These PB NPs exhibit excellent anti-oxidant properties, making them effective at regulating inflammation and promoting wound healing [236].

Our intention here is to highlight approved nanomaterials that exhibit nanozyme properties. In conclusion, the ongoing research and approval of nanomaterials, particularly those exhibiting enzyme-like activities and anti-oxidant properties, highlight the immense potential for developing novel, effective treatments for wound healing and infection management. As these materials continue to evolve, they offer promising new avenues for advancing wound care therapies.

## Challenges and prospects

This review explores the rapid advancements in biotechnology and nanotechnology that have led to the development of diverse nanozyme-based wound dressings. We provide a detailed analysis of the multifaceted enzyme-like activities of nanozymes and their mechanisms underlying chronic wound healing, along with an in-depth examination of their preparation strategies. We also highlight the latest research breakthroughs using these dressings to treat infected and diabetic wounds. Despite promising progress, significant challenges and unresolved issues still need to be addressed to fully harness the therapeutic potential of nanozymes in wound care.

1. The development of nanozyme-based dressings with multienzyme-like activities and multifunctionalities shows exciting promise given the growing demand for chronic wound treatment. However, integrating multiple enzyme-like activities within a single nanostructure presents a significant challenge, as it requires not only the precise combination of different catalytic functions but also the ability to maintain their stability and activity under varying environmental conditions. For example, balancing the interactions between different enzyme activities to avoid the enhancement of one enzyme activity leading to the weakening of another is a key issue. This integration demands innovative approaches to ensure that distinct enzyme-like properties can co-exist without interference and remain effective over extended periods, especially in complex biological or industrial settings. To address this, research should focus on hierarchical nanostructure design, which could compartmentalize different catalytic activities, minimizing undesired cross-interactions. Furthermore, the use of machine learning to predict optimal enzyme activity combinations and configurations could accelerate innovation in this area [237].
2. The enzyme-like activity of nanozymes plays a crucial role in regulating oxidative stress during chronic wound treatment. Currently, most studies primarily focus on the changes in ROS, which oversimplifies the underlying mechanisms of nanozyme catalysis in chronic wound healing [238–240]. Nanozymes not only mitigate oxidative stress through antioxidation but also engage in multiple catalytic pathways, including ROS balance regulation, cell signaling modulation, and immune microenvironment optimization. For instance, MnO_2_ nanozyme-loaded can simulate the activities of SOD and CAT for synergistic catalysis, forming a cascade reaction to reduce oxidative stress and accelerate wound healing [241]. Zn-dihydromyricetin NPs down-regulate excessive intracellular glucose and hexokinase two levels, inhibit excessive glycolysis, and counteract glycation-induced cellular dysfunction [242]. In addition, the release of low concentrations of Cu^2^⁺ by copper-based nanozymes can activate the anti-inflammatory signal pathway, induce macrophages to transform from M1 type to M2 type, inhibit chronic inflammation, and promote collagen deposition [243]. These mechanisms highlight that nanozyme catalysis extends beyond simple ROS clearance, offering a multi-faceted approach to optimizing the wound microenvironment. Future research should focus on the mechanism of specific catalytic pathways and offer a fresh perspective for improving nanozyme-based treatments for chronic wounds.
3. Excessive levels of reduced nicotinamide adenine dinucleotide, reduced nicotinamide adenine dinucleotide phosphate, and GSH-induced reduction stress are as harmful as oxidative stress [244], which will destroy the redox balance. In chronic wounds, vascular damage often leads to hypoxia, which exacerbates oxidative stress and impairs healing. Under anoxic conditions, the absence of hydrogenases 2 and 3 increases the nicotinamide adenine dinucleotide/NAD⁺ ratio, providing more electrons for the single-electron reduction of oxygen to O₂, thereby elevating mitochondrial ROS levels [245, 246]. Additionally, hypoxia induces an increase in cellular GSH levels through both HIF-1α-dependent and -independent pathways, potentially leading to cytotoxic effects that may hinder wound healing [247, 248], which is presumed unfavorable to wound healing. Furthermore, reductive stress has been linked to chronic wound complications, including diabetes, as it can disrupt cellular metabolism and function [249]. Given these factors, it is plausible that managing reductive stress could play a critical role in promoting chronic wound healing. Previous research has shown that nanozymes with dehydrogenase-like activity can accelerate dehydrogenation processes in cell metabolism, leading to the excessive accumulation of reducing species and triggering reduction stress [250]. Therefore, nanozyme-based wound dressings hold significant potential for addressing reduction stress and offer a novel strategy for wound treatment. However, it is crucial to develop strategies that carefully balance reduction stress and oxidative stress to avoid their adverse biological consequences.
4. Smart nanozyme dressings can adapt their functions based on changes in the wound microenvironment, offering personalized treatment. By combining these dressings with wearable devices for real-time monitoring of parameters such as temperature, humidity, and inflammation markers, dressing performance can be dynamically adjusted, enhancing therapeutic outcomes and patient compliance. However, the integration of this system is technically complex. Therefore, studying biosensor materials that seamlessly integrate with nanozyme systems is crucial. The development of a modular platform that connects nanozymes with flexible electronics can streamline integration, enhance scalability, and optimize performance. Moreover, when designing intelligent nanozyme dressings, it is essential to thoroughly understand their clinical needs, align them with real-world application scenarios, and enhance the practicality of the product.
5. In terms of translational application, early consideration of the large-scale production and biosafety of nanozyme products is essential. Compared with natural enzymes, nanozymes offer the advantages of ease of large-scale production and lower costs. However, for nanozyme-based dressings, a major challenge lies in developing streamlined industrial processes for both nanozyme synthesis and the assembly of the dressing matrix. These processes must not only ensure a high loading efficiency of nanozymes but also effectively reduce production costs. The metabolic processes of nanozymes in the human body and their interactions with tissues remain unclear, raising concerns about their biocompatibility. Furthermore, ensuring the long-term stability of nanozymes in the wound environment, as well as the safety of their degradation byproducts, presents a critical challenge, involving the control of material stability and degradation behavior under complex physiological conditions. In-depth pharmacokinetic and toxicological studies using advanced imaging and omics technologies can provide a clearer understanding of nanozyme behaviors in vivo. Collaborative efforts to standardize regulatory frameworks will also facilitate smoother clinical translation.
6. Current nanozyme delivery methods, such as injections, microneedle patches, and nanoparticle suspensions, have limitations, including pain, insufficient drug loading, and short residence times on the wound surface. Furthermore, the removal of dressings adhering to the wound surface can potentially damage granulation tissue, thereby impeding the healing process. Therefore, novel delivery systems are urgently needed to achieve precise release, multifunctionality, painless removal, and enhanced biocompatibility, thereby improving therapeutic outcomes and meeting the demands of chronic wound care. Promising approaches, including the development of stimuli-responsive hydrogels capable of prolonged release and bioadhesive dressings, will improve wound adhesion. Furthermore, 3D printing and bioprinting technologies could enable the creation of patient-specific and multifunctional nanozyme delivery systems [251].
7. Despite extensive research on the clinical translation of nanozyme wound dressings, standardized preclinical trials remain a key challenge. Ensuring safety and efficacy requires a comprehensive preclinical evaluation. For nanozyme activity detection, it is recommended to develop cell-level assays that better mimic clinical scenarios. For instance, labeling ROS with fluorescent probes enables real-time visualization of intracellular ROS dynamics regulated by nanozymes, providing a clearer assessment of their therapeutic effects [252]. Moreover, precise control of key parameters, such as cell source selection, 3D tissue model construction, and cell state regulation based on specific characteristics (e.g., high glucose, hypoxia, infection). It is essential for accurate in vitro simulation of the wound microenvironment [253]. For in vivo studies, standardized wound models (e.g., diabetic ulcers, burns, chronic wounds) should be established to systematically assess healing rate, tissue regeneration, inflammation, and long-term toxicity [254]. Advanced techniques, such as single-cell sequencing and transcriptomics, can further elucidate the mechanisms of nanozyme action. Establishing standardized protocols and conducting multi-center validation will enhance data reproducibility and improve clinical translation reliability.

## Conclusions

In conclusion, nanozyme-driven multifunctional dressings offer a novel approach to chronic wound care. By actively modulating the wound microenvironment, these dressings surpass the limitations of conventional therapies through their multifunctional and adaptable design. However, current research predominantly focuses on small-scale laboratory experiments. To bridge the gap between research and clinical application, future efforts should emphasize interdisciplinary collaboration, fostering a seamless transition from understanding the underlying mechanisms to clinical implementation. A key challenge lies in balancing effective treatment with cost control and ensuring high patient acceptance. Ethical and regulatory considerations, including biocompatibility and long-term safety, must remain central to development efforts. Looking ahead, the next generation of nanozyme-driven dressings should incorporate real-time adaptability to the dynamic wound microenvironment, leveraging intelligent biomaterials and responsive drug delivery strategies. With continued technological innovation and robust clinical validation, these advanced dressings have the potential to redefine chronic wound management, ultimately enhancing patient outcomes and broadening access to superior care.

## Data Availability

No applicable.

## Abbreviations

CAT: Catalase
3D: Three-dimensional
DFUs: Diabetic foot ulcers
ECM: Extracellular matrix
GOx: Glucose oxidase
GSH: Glutathione
H_2_O_2_: Hydrogen peroxide
HIF-1α: Hypoxia-inducible factor-1α
IL: Interleukin
MMPs: Matrix metalloproteinases
NPs: Nanoparticles
NSs: Nanosheets
O_2_: Oxygen
·OH: Hydroxyl radicals
PB: Prussian blue
PDA: Polydopamine
PTT: Photothermal therapy
POD: Peroxidase
PVA: Polyvinyl alcohol
ROS: Reactive oxygen species
SOD: Superoxide dismutase
TNF: Tumor necrosis factor
VEGF: Vascular endothelial growth factor
